# Daily Estimation of Global Solar Irradiation and Temperatures Using Artificial Neural Networks through the Virtual Weather Station Concept in Castilla and León, Spain

**DOI:** 10.3390/s22207772

**Published:** 2022-10-13

**Authors:** Francisco J. Diez, Ouiam F. Boukharta, Luis M. Navas-Gracia, Leticia Chico-Santamarta, Andrés Martínez-Rodríguez, Adriana Correa-Guimaraes

**Affiliations:** 1 Department of Agricultural and Forestry Engineering, University of Valladolid, Campus La Yutera, 34004 Palencia, Spain; 2 International Department, Harper Adams University, Newport TF10 8NB, UK

**Keywords:** daily global solar irradiation, daily maximum temperature, daily average temperature, daily minimum temperature, evapotranspiration, agro-meteorology, Artificial Neural Networks (ANNs), Virtual Weather Station (VWS) concept, spatial interpolation

## Abstract

In this article, the interpolation of daily data of global solar irradiation, and the maximum, average, and minimum temperatures were measured. These measurements were carried out in the agrometeorological stations belonging to the Agro-climatic Information System for Irrigation (SIAR, in Spanish) of the Region of Castilla and León, in Spain, through the concept of Virtual Weather Station (VWS), which is implemented with Artificial Neural Networks (ANNs). This is serving to estimate data in every point of the territory, according to their geographic coordinates (i.e., longitude and latitude). The ANNs of the Multilayer Feed-Forward Perceptron (MLP) used are daily trained, along with data recorded in 53 agro-meteorological stations, and where the validation of the results is conducted in the station of Tordesillas (Valladolid). The ANN models for daily interpolation were tested with one, two, three, and four neurons in the hidden layer, over a period of 15 days (from 1 to 15 June 2020), with a root mean square error (RMSE, MJ/m^2^) of 1.23, 1.38, 1.31, and 1.04, respectively, regarding the daily global solar irradiation. The interpolation of ambient temperature also performed well when applying the VWS concept, with an RMSE (°C) of 0.68 for the maximum temperature with an ANN of four hidden neurons, 0.58 for the average temperature with three hidden neurons, and 0.83 for the minimum temperature with four hidden neurons.

## 1. Introduction

Agricultural productivity can be increased by knowing and predicting more precisely crop yields under various conditions. This is a key concept in both precision agriculture and agricultural modelling. Several authors have studied the different techniques applied in precision agriculture and in the modelling of crop production where they involve meteorological variables, with the objective of improving quality, profitability, resource use efficiency and sustainability [1,2,3]. Among these techniques, the application of variable doses of water, fertilizers and agrochemicals (while considering agrometeorological conditions), as well as the estimation of production (based on the evolution of meteorological variables and the physiological response of crops), are the most frequently used and are currently adopted by many farmers. Indeed, in most cases, crop recommendations are based on data recorded from field studies that compile their conditions (soil and environment) [4].

The impact of global solar irradiation on the Earth’s surface has a significant influence on a country’s economy, including, for example, agricultural productivity, renewable energy use, food security and human health risks [5], as reported in [6,7,8,9,10].

Prediction and estimation studies of meteorological variables focus on measured data as inputs to the model. Franco et al. [11] found that there is a lack of such studies that use ANN models, and that focus on generating data in sites where such data are not available, so that they can be used as inputs to other models.

Solar radiation is a fundamental factor for most physical and biophysical processes due to its role contributing in to the balance of energy and water. However, interpolation techniques are applied to large areas and do not capture the high variation at finer scales. Fu and Rich [12] calculated insolation maps based on regression analysis of atmospheric conditions, elevation, surface orientation and the influence of surrounding topography, by correlating ground temperature with insolation and elevation, explaining the marginal variation of other factors, such as crop canopy, in the vicinity of Rocky Mountain Biological Laboratory, Gunnison, CO, USA, which area is approximately 300 km^2^ and has dramatic topographic variation, with an elevation ranging from 2500 to 4300 m.

The lack of site-specific global solar radiation data is a significant barrier to most applications of crop models. Indeed, Mavromatis and Jagtap [13] evaluated several empirical methods for estimating daily solar radiation from observed maximum and minimum air temperatures, using data from urban and rural sites in Florida (USA), and using spatially interpolated coefficients to improve the results, which are applied to estimate crop yield potential and evapotranspiration. The Donatelli–Bellocchi model [14,15] achieved the most accurate estimates with a Root Mean Square Error (RMSE) of 3.1–4.1 MJ/(m^2^ d) in rural areas and 3.2–4.9 MJ/(m^2^ d) in urban areas.

Spatial interpolation is a classical geostatistical operation that aims to predict values assigned to unobserved locations from a defined sample of data on specific substrates. However, the underlying continuity and heterogeneity of spatial data are too complex to be approximated by traditional statistical models. By using deep learning models, in particular the idea of conditional generative adversarial networks (CGAN) [16], deeper representations of sampled spatial data and their interactions with local structural patterns can be captured. Zou et al. [17], with a case study (global solar radiation) on elevations in southeast of China, demonstrated the model ANN capacity to achieve outstanding interpolation results compared to the benchmark methods: a model ANN (9-17-1) provided better accuracy (RMSE = 1.34 MJ/m^2^, and R^2^ = 0.91) compared to the improved Bristow–Campbell model (RMSE = 2.19 MJ/m^2^, and R^2^ = 0.83) and the improved Ångström–Prescott model (RMSE = 2.65 MJ/m^2^, and R^2^ = 0.68).

Environmental variables are recorded by point sampling. However, precision agriculture requires more precise and specific knowledge of these characteristic variables near or within the crop, and thus, spatially continuous data on environmental variables becomes necessary. Li and Heap [18] classified 25 Spatial Interpolation Methods (SIM) into three different categories: non-geostatistical, geostatistical, and combined methods, and provided guidelines and suggestions for selecting the appropriate method for a specific environmental dataset.

A typical spatial interpolation method, which is very efficient and simple, is Inverse Distance Weighting (IDW), for which Li et al. [19] proposed a new approach, called Dual IDW (DIDW), which takes into account the correlation of the data, to avoid unfavourable estimates with unevenly distributed samples. A case study based on Walker Lake data indicates that DIDW significantly improves interpolation accuracy over traditional IDW, and also slightly outperforms Ordinary Kriging (OK) for small data samples to capture adequate spatial continuity.

The spatial interpolation of the Earth’s weather variables occupies an important role in climate studies, but most of the traditional spatial interpolation methods do not consider geographical semantics in their practical application. Wu et al. [20] proposed an improved algorithm for IDW by considering geographic Semantics (SIDW), which adds the influence of land use type on the interpolation of land surface temperature data by the Landsat 8 OLI-TIRS satellite over China, achieving generally higher accuracy and precision than IDW, Kriging, natural neighbour, and spline function interpolation methods.

Loghmari et al. [21] developed and evaluated two monthly spatial interpolation models of global solar radiation, for the purpose of predicting global solar radiation within a distance of more than 50 km in southern and central Tunisia: an artificial neural network (ANN) that obtained better results than a model based on IDW.

In order to spatially fill gaps (nowcasting) in micrometeorological data sets (wind, humidity and temperature), Gunawardena et al. [22] employed Multivariate Linear Regression (MLR) and ANN at eight locations, using measurements from three nearby weather stations, covering scales from 100 m to 5 km. These measurements were made in regions marked by complex terrain, where spatial variability is high on small length scales, which in this case is the Cadarache Valley, which is located in southeastern France, from December 2016 to June 2017, demonstrating that both methods are acceptable.

In this case [23], it is notable the interpolation of the observed weather in the centre of a 25 by 25 km grid, where the weather data is homogeneous, and the temperature, sunshine, humidity and wind speed are expected to change gradually at distances of 50 to 150 km in the European Commission’s MARS (Monitoring Agriculture with Remote Sensing) Crop Yield Forecasting System (MCYFS) wiki.

Geographic Information Systems (GIS) offer different options to analyze and represent the spatial heterogeneity of the incident solar radiation in a given area. Martín and Dominguez [24] presented a description of the methods for estimating the distribution of solar radiation in geographical areas, from a sample of data, using deterministic techniques (global polynomial interpolation, local polynomial interpolation, inverse distance weighting and radial basis functions) and geostatistical techniques (kriging and co-kriging) applying them for the summer solstice 2011, from 45 stations in Spain. Indeed, the global polynomial method presents interpolations closer to the real value, the geostatistical methods, in turn, generally present very low squared errors (the universal kriging and the ordinary co-kriging are those that show the best adequacy in the results).

The data, which is collected at discrete weather stations, can only be meaningful when represented by surfaces. Spatial interpolation methods help to convert the point data into surfaces by estimating missing values for areas where data is not collected. In addition to the objective, the total number of data points, their location and their distribution in the study area affect the accuracy and efficiency of the interpolation. Keskin et al. [25] aimed to investigate the optimal spatial interpolation method for mapping meteorological data (precipitation, temperature and wind speed) in the Northern part of Turkey, using the interpolation methods (IDW, kriging, radial basis and natural neighbour). This investigation was carried out in January 2005, resulting in a three-locations average RMSE for a temperature of 0.94 °C with IDW, 0.75 °C with kriging and 0.70 °C natural neighbour.

Yazar [26] performed spatial interpolation of solar radiation with data from 81 agrometeorological stations over heterogeneous agricultural areas including different crop species, irrigation techniques, and topographical and other conditions in Southeastern Turkey, by applying Ordinary Kriging (OK) individually and to reduce the Ordinary Co-Kriging (OCK) error with solar radiation related data (air temperature, vapour pressure deficit and digital elevation model), with up to 21% accuracy, which allowed for better evaluation and management of crop development and yield.

Leirvik and Yuan [5] employed statistical methods (Random Forest (RF); Linear Regression (LR); Generalized Additive Regression (GAM); Least Squares Dummy Variable (LSDV); Ordinary Kriging (OK); and combinations, as LR + OK, GAM + OK, and LSDV + OK) to interpolate missing values in a monthly dataset spanning nearly five decades of global solar irradiation over the Earth’s surface, highlighting the benefits of using Machine Learning in environmental research.

Antonić et al. [27] used ANN models for monthly mean values of meteorological variables (air temperature, daily minimum and maximum air temperature, relative humidity, precipitation, global solar irradiation and evapotranspiration) through data obtained from 127 meteorological stations in Croatia. The inputs used (elevation, latitude, longitude, month and time series of the respective climatic variables) were from two meteorological stations. The quality of the results allows the construction of spatial distributions of the average climate for a given period, which would be useful for dendroecological analysis.

Siqueira et al. [28] performed the generation of synthetic daily solar irradiation series from spatial interpolation based on ANNs, employing geographic variables (latitude, longitude and altitude) and meteorological variables (precipitation, maximum and minimum temperature), which were easily available. The data were measured during the months of November (from 2001 to 2006) over seven locations in Pernambuco, Brazil. 

Many climate studies need to generate predictions of a climate variable at a given location using values from other locations. Snell et al. [29] conducted a spatial interpolation of daily maximum surface air temperatures using ANNs, so as to generate estimates at 11 locations in the central U.S. continent, using information from a network of surrounding stations for the 4- and 16-point cases and over a 63-year period (from 1931 to 1993) that were used as input and output vectors for the ANNs. The results obtained are better than the spatial average, nearest neighbour and inverse distance methods, and the potential of using ANNs for downscaling General Circulation Models (GCMs) of temperature is discussed.

Rigol et al. [30] performed a spatial interpolation of daily minimum air temperature using an ANN trained with input variables (date, field variables and neighbouring temperature observations) for a full year, covering an area of 100 km × 100 km in Yorkshire, UK, analyzing the internal weights of the inputs to estimate the degree of spatial correlation between neighbouring stations, and the most influential variables contributing to the trend. The performance when testing ANN (33-1-1) is RMSE = 3.15 °C, of ANN (19-4-1) is RMSE = 1.26 °C, and of ANN (45-4-1) RMSE = 1.15 °C.

Zambon et al. [31] reviewed Industry 4.0 procedures suitable for the agricultural sector, while pointing out that the 4.0 revolution in agriculture is still limited to a few innovative companies. Additionally, environmental variability and stochastic events contribute to a high degree of uncertainty in the supply chain and a lack of predictability in agricultural operations. This is where recent technologies related to the digital age, such as precision agriculture, which uses positioning technologies combined with the application of sensors and data, provide digital information in all agricultural processes.

In this paper, the concept of a Virtual Weather Station (VWS) is used and employs meteorological data from real stations to estimate data from a nearby location that does not have a weather station. As part of the VWS development, the performance of ANN models for interpolating each separate meteorological variable (global solar irradiation, maximum, average and minimum temperatures) was evaluated. The performance of the models is compared with those obtained by Franco et al. [11], who proposed the use of a VWS in places where meteorological data are needed, as an alternative to their acquisition, when it is not possible to install a meteorological station. The ANN models, in this case, were used with all the variables of the same place, while in this article, the estimation of each variable (solar irradiation and temperatures) is carried out separately (an ANN model for each meteorological variable).

## 2. Materials and Methods

In this section, the following points are described: (1) the meteorological data used with the tested geographic interpolation models, corresponding to global daily solar irradiation and ambient temperature (maximum, average and minimum), as well as information on the location of the agro-meteorological stations where these data were recorded; (2) the ANN models designed for the estimation of the analyzed meteorological variables; and (3) the statistics used to analyze the accuracy of the results obtained by the ANN-based interpolation models that have been examined.

### 2.1. Daily Data on Global Solar Irradiation and Ambient Temperature (Maximum, Average and Minimum)

**Figure 1:**
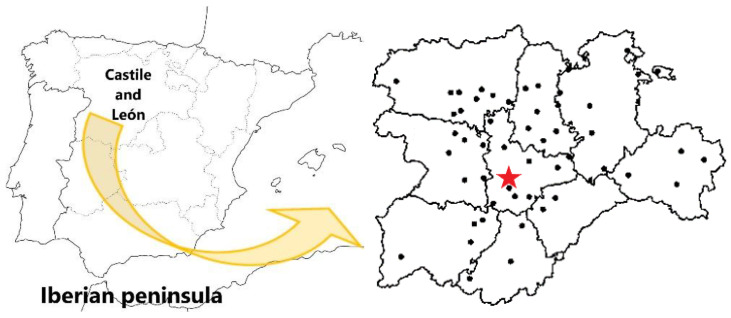
Location of the 54 agrometeorological stations belonging to the Agro-climatic Information System for Irrigation (SIAR) located in Castilla and León Region, Spain [32], highlighting (red star) the site of the agrometeorological station referenced for this study (Tordesillas, Valladolid).

The daily average data of global solar irradiation and ambient temperature (maximum, mean and minimum) used in this article, for a 15-day period (from 1 to 15 June 2020), were collected in the 54 agrometeorological stations (Appendix A) belonging to the Agro-climatic Information System for Irrigation (SIAR, Sistema de Información para el Asesoramiento al Riego, in Spanish), located in Castilla and León Region, in the North-central part of Iberian Peninsula, as shown in the map presented in Figure 1 and in Table A1 (data of altitude, latitude and longitude).

SIAR is a project financed by the Ministry of Environment and Rural and Maritime Areas of Spain, which is managed by the Agricultural Technological Institute of Castilla and León, (ITACyL, Instituto Tecnológico Agrario de Castilla y León, in Spanish), through the Meteorological Information Service [32]. The SIAR project helps farmers to manage irrigation water in an optimal way, advising them on the doses to be applied at each time of the year, depending on the phenological stage of the crop, by calculating the reference evapotranspiration (ETo).

Within the agrometeorological stations of the SIAR network, solar irradiance is measured by a Skye SP1110 pyranometer (Campbell Scientific, Inc., North Logan, UT, USA), consisting of a silicon photocell sensitive to radiation between 350 and 1100 nm, while the ambient temperature is measured by a Pt-1000 temperature sensor, which is based on the variation of platinum resistance with temperature. The linearization and amplification electronics for these sensors are located next to a Vaisala HMP45C probe (Campbell Scientific, Inc., North Logan, UT, USA), which is used to measure ambient temperature and relative humidity, in the temperature ranges of −40 to 60 °C, and 0 to 100%, respectively.

The climatic classification for the location of most agrometeorological stations is Csb, with some located in areas classified as Cfb, Csa and BSk types [33], according to the Koppen-Geiger climate classification.

### 2.2. Estimation of Solar Irradiation and Ambient Temperature Using Artificial Neural Networks

**Figure 2:**
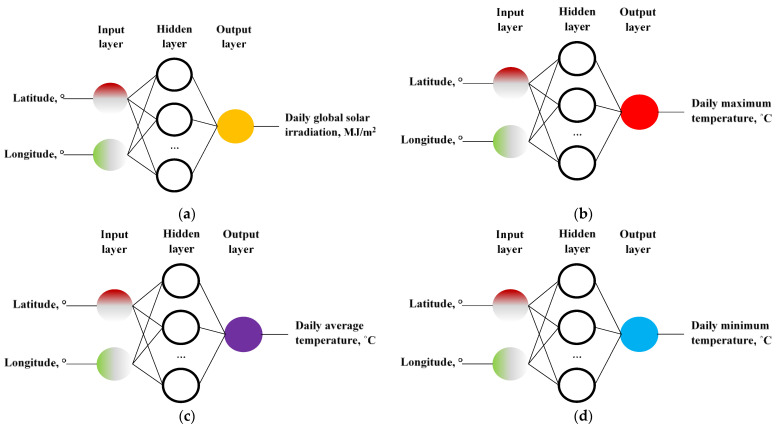
The architecture of the models evaluated with Artificial Neural Networks (ANN). Layers (input-hidden-output) (2-1…4-1) based on the input variables [latitude and longitude] to individually estimate: (**a**) daily global solar irradiation; (**b**) daily maximum temperature; (**c**) daily average temperature; (**d**) daily minimum temperature.

The architectures of the ANNs used for the evaluated geographic interpolation models are illustrated in Figure 2. All of them contain two inputs (longitude and latitude) and one output, which can be the daily global solar irradiation, or the daily mean values of the ambient temperature (maximum, average, or minimum).

The implementation of the ANNs was performed in MATLAB Software with the *feedforwardnet* function, dimensioned with the input and output data vectors, which determine the size of the respective layers, generating a Multilayer feed-Forward Perceptron (MLP) type ANN with a single hidden layer, where the selected activation function between neurons in the hidden layer was the hyperbolic sigmoidal tangent (*tansig*), while the selected transfer function for the neurons in the output layer was linear (*purelin*). The Levenberg–Marquardt back-propagation (BP-LM) algorithm was applied to achieve fast optimization (*trainlm*) [34,35].

The training of the ANNs was performed with the *train* function, with matrices of input and output data vector, carried out daily in 53 agrometeorological stations of the SIAR network (all of them belonging to this network, except the agrometeorological station of Tordesillas, used in the validation phase of the results), over a period of 15 days (from 1 to 15 June 2020). Finally, the *sim* function was used, testing the ANNs previously trained with 1, 2, 3, and 4 neurons in the hidden layer, to estimate each meteorological variable studied separately, over the same 15 days at the station located in Tordesillas (Valladolid, Figure 1), with geographic coordinates 41°30′32″ N and 4°59′20″ W, altitude 658 mamsl, used as reference for the validation. The period from June 1 to 15 was chosen because it is the period of the year when agricultural activity is the highest in the Iberian Peninsula, coinciding with the end of winter crops and the beginning of summer crops.

### 2.3. Statistics for the Validation of the ANN Models 

The accuracy of the results obtained by the ANN models in the validation phase was analyzed using the following statistics: Root Mean Square Error (RMSE, solar irradiation MJ/m^2^ and temperature °C), using Equation (1); and the coefficient of determination (R^2^), as an indicator of the level of model fit, using Equation (2).

(1) $$\mathrm{RMSE}=\sqrt{\frac{\sum_{i=1}^{n}{\left(Y_{i}-{\hat{Y}}_{i}\right)}^{2}}{n}}$$

(2) $$R^{2}=1-\frac{\sum_{i=1}^{n}{\left(Y_{i}-{\hat{Y}}_{i}\right)}^{2}}{\sum_{i=1}^{n}{\left(Y_{i}-\bar{Y}\right)}^{2}}$$

## 3. Results

This section presents the results obtained by the ANN models for the daily estimation of global solar irradiation (1) and ambient temperature (maximum (2), average (3), and minimum (4)) at the agrometeorological reference station SIAR, located in Tordesillas, Valladolid, Castilla and León, Spain.

### 3.1. ANN Models for Estimating Daily Global Solar Irradiation at the Reference Station

The results of the ANN models for estimating daily global solar irradiation at the reference station presented in Figure 2a are shown in Table 1. The best result is obtained when using ANN (2-4-1) with RMSE = 1.04 MJ/m^2^, which improves on the best ANN result of Franco et al. [11] for the summer months of 1.63 MJ/m^2^, by using the rectified linear unit activation function.

### 3.2. ANN Models for the Estimation of the Maximum Daily Temperature in the Reference Station

The results of the ANN models shown in Figure 2b for the estimation of the daily maximum temperature at the reference station, are presented in Table 2. The best result obtained is the ANN (2-4-1) with RMSE = 0.68 °C, which improves the best result of the ANNs Franco et al. [11] for the summer months by 1.28 °C using the sigmoid activation function.

### 3.3. ANN Models for the Estimation of the Average Daily Temperature in the Reference Station

The results of the ANNs models shown in Figure 2c for estimating the daily mean temperature at the reference station are presented in Table 3. The best result is obtained by ANNs (2-3-1) with RMSE = 0.58 °C, which improves the best ANN performance Franco et al. [11] for the summer months by 0.99 °C when using the hyperbolic tangent activation function.

### 3.4. ANN Models for the Estimation of the Minimum Daily Temperature in the Reference Station

The results of the ANN models shown in Figure 2d for the estimation of the daily minimum temperature at the reference station, are visualized in Table 4. It obtained the best result for the ANN (2-4-1) with RMSE = 0.83 °C, which improves the best result of all ANNs Franco et al. [11] for the summer months by 1.55 °C, when using the hyperbolic tangent activation function.

## 4. Discussion

In this paper, ANNs were used to perform spatial weather forecasts using data measured by SIAR agrometeorological stations in Castilla and León (Spain), one of the largest regions in Europe (94,224 km^2^, where more than half of the area is agricultural land), using meteorological data from both the area near the reference station and the neighbouring areas, which achieved a better performance of the ANN models. Loghmari et al. [21] applied an ANN model using the available meteorological data in the target area with a Recorded Average Relative Root Mean Square Error (ARRMSE) of 6.4%, while the IDW model estimated the global solar radiation measured in nearby areas with an error of 5.11%.

The date set used by Franco et al. [11] to interpolate the values of the most important meteorological variables in agriculture using an ANN was daily precipitation (mm), evapotranspiration ETo (mm), mean daily air temperature (°C), maximum temperature (°C), minimum temperature (°C), mean daily relative humidity (%), maximum relative humidity (%), minimum relative humidity (%), mean wind speed (m/s) and total solar irradiation (MJ/m^2^) during the summer months (June, July and August) by the same SIAR agrometeorological stations in the territory of Castilla and León, Spain.

In this paper, ANN models are performed independently for each daily variable studied (global solar irradiation, and maximum, average and minimum temperatures) from the geographic coordinates [longitude and latitude] of the location to be estimated, achieving better performance in RMSE values (1.04 MJ/m^2^, 0.68 °C, 0.58 °C, and 0.83 °C, respectively), compared to the ANN models. Franco et al. [11] simultaneously analyzed in the same ANN, ten meteorological variables, during the summer months, obtaining RMSE values of 1.63 MJ/m^2^, 1.28 °C, 0.99 °C, and 1.55 °C, respectively, for the same variables.

## 5. Conclusions

Precision agriculture can improve the performance of crops, and thus increase agricultural productivity, by considering a precise knowledge of the meteorological variables that affect them in their development. The number of agrometeorological station networks is increasing, but it is still interesting to have data from the specific location of the crops, which can be obtained by interpolating the data measured by the agrometeorological station network. Strong et al. [36] assessed and evaluated the barriers to the adoption of smart agriculture through the Internet of Things (IoT) among Brazilian farmers in the Rio Grande do Sul, where they found that elements such as compatibility, complexity, testability, and visibility were the predictors of farmers’ adoption of innovative solutions. As for ANN models, they were analyzed in this paper to describe the importance of their application for the adoption of climate-smart agriculture.

Kilelu et al. [37] carried out a report on the development of enterprises providing agricultural services in the context of the transformation of agricultural value chains and food systems in the dairy sector in Kenya, where they have the potential to provide innovation support to entrepreneurial farmers as well as contribute to the sustainable growth of the sector.

In this article, ANN models were used to interpolate the data measured daily by the SIAR network of agrometeorological stations in the Region of Castilla and León (Spain) for several meteorological variables: global solar irradiation, maximum, average and minimum temperatures, from the geographical coordinates of the location where the interpolation was carried out, by means of an ANN model for each of the variables studied. This study uses meteorological data available in the target region (areas close to the reference station) and in neighbouring regions (areas far from the reference station). The possibility of having synthetic meteorological data that best represent the local meteorology at each place and time is therefore very important to be able to apply advanced agricultural forecasting techniques that, for example, are related to the knowledge of the phenological behaviour of plants of productive interest, to the prediction of the necessary irrigation doses and the incidence of pests and diseases, or to the estimation of the potential product of the crops [38,39,40].

The results obtained from this study are more successful than those obtained previously for the same SIAR network by applying a single ANN model for all meteorological variables (10 variables). The key to this improvement in results is the use of more simplified and simpler ANN models, which provide a more accurate ANN (Occam’s razor).

In addition, the results obtained from the VWS in this study can be applied to make the prediction, at the same location, of the global solar irradiation of the next day with the ANN models developed by Diez et al. [34], and to estimate the hourly distribution of the ambient temperature, during the 24 h of the day, with the ANN models developed by Diez et al. [35], as well as the prediction of the values, for the next day, of the temperature (maximum, average and minimum).

Future studies that develop these ANN models for the interpolation of meteorological variables from geographic coordinates for crop production could include a predictor variable that directly affects the variable to be estimated (in a sloping terrain, its orientation to interpolate solar irradiation, or in the case of temperatures, the type of vegetation cover) that would increase the accuracy of the ANN models.

## Figures and Tables

**Table 1:** Daily global solar irradiation (MJ/m^2^) in Tordesillas (Valladolid) measured for 15 days (i.e., 1–15 June 2020), estimated with the neural architectures varying the number of neurons from four to one in the hidden layer (i.e., ANN (2-4-1), ANN (2-3-1), ANN (2-2-1) and ANN (2-1-1)), and fitting of the statistics.

Tordesillas	Data	ANN (2-4-1)	ANN (2-3-1)	ANN (2-2-1)	ANN (2-1-1)
1 June 2020	27.58	26.85	27.27	27.62	26.83
2 June 2020	25.61	25.60	26.03	25.79	25.79
3 June 2020	24.38	23.16	22.23	21.90	22.77
4 June 2020	27.74	25.29	25.07	24.68	24.67
5 June 2020	31.09	30.41	30.79	29.92	30.00
6 June 2020	27.45	27.29	26.22	26.29	25.98
7 June 2020	17.94	17.58	16.14	16.92	17.10
8 June 2020	26.96	26.75	26.72	26.60	26.47
9 June 2020	24.94	26.89	27.32	27.06	26.07
10 June 2020	28.46	27.96	28.60	27.73	27.64
11 June 2020	21.55	20.72	22.33	21.74	21.07
12 June 2020	14.93	14.16	15.71	15.94	14.41
13 June 2020	21.29	20.51	21.10	20.21	20.36
14 June 2020	27.64	26.34	26.60	26.31	26.18
15 June 2020	22.21	22.30	23.11	22.43	22.69
RMSE		1.04	1.31	1.38	1.23
R^2^		0.94	0.90	0.89	0.91

RMSE, root mean square error (MJ/m^2^); R^2^, determination coefficient. The best results are underlined.

**Table 2:** Daily maximum temperature (°C) in Tordesillas (Valladolid) measured for 15 days (i.e., 1–15 June 2020), estimation performed with the neural architectures varying the number of neurons from four to one in the hidden layer (i.e., ANN (2-4-1), ANN (2-3-1), ANN (2-2-1) and ANN (2-1-1)), and fitting of the statistics.

Tordesillas	Data	ANN (2-4-1)	ANN (2-3-1)	ANN (2-2-1)	ANN (2-1-1)
1 June 2020	28.73	27.92	27.96	27.72	27.54
2 June 2020	29.73	29.01	29.34	29.05	28.57
3 June 2020	27.73	26.52	26.17	26.18	25.58
4 June 2020	21.26	20.98	20.78	21.09	20.82
5 June 2020	26.86	26.60	26.28	26.68	26.30
6 June 2020	27.13	26.12	26.48	25.92	25.59
7 June 2020	19.19	18.15	19.26	18.57	18.74
8 June 2020	20.06	20.05	19.86	19.91	19.89
9 June 2020	20.26	20.26	20.60	21.02	20.62
10 June 2020	24.8	24.33	24.14	24.11	24.12
11 June 2020	21.46	20.66	20.42	20.32	20.39
12 June 2020	18.2	17.45	16.71	16.84	16.42
13 June 2020	18.99	19.37	19.30	19.45	19.28
14 June 2020	21.79	21.98	21.18	21.29	21.17
15 June 2020	22.79	22.20	22.17	22.00	22.22
RMSE		0.68	0.77	0.86	1.04
R^2^		0.97	0.96	0.95	0.92

RMSE, root mean square error (°C); R^2^, determination coefficient. The best results are underlined.

**Table 3:** Daily average temperature (°C) in Tordesillas (Valladolid) measured for 15 days (i.e., 1–15 June 2020), estimation performed with the neural architectures varying the number of neurons from four to one in the hidden layer (i.e., ANN (2-4-1), ANN (2-3-1), ANN (2-2-1) and ANN (2-1-1)), and fitting of the statistics.

Tordesillas	Data	ANN (2-4-1)	ANN (2-3-1)	ANN (2-2-1)	ANN (2-1-1)
1 June 2020	20.39	19.71	19.40	19.47	19.26
2 June 2020	22.00	21.47	21.53	20.98	20.57
3 June 2020	19.04	18.51	18.20	18.40	17.98
4 June 2020	16.15	16.02	15.42	14.92	15.35
5 June 2020	16.83	16.13	16.50	16.80	16.57
6 June 2020	18.09	17.46	18.04	17.53	17.31
7 June 2020	14.65	13.61	13.61	13.62	13.96
8 June 2020	13.77	13.03	13.14	12.94	12.82
9 June 2020	13.83	13.06	13.83	13.09	13.05
10 June 2020	16.68	15.75	16.20	15.75	15.83
11 June 2020	15.11	14.69	14.50	14.80	14.02
12 June 2020	11.88	11.31	11.88	11.18	11.20
13 June 2020	13.45	13.37	13.15	12.84	12.72
14 June 2020	15.43	15.10	15.10	14.76	14.59
15 June 2020	15.99	15.84	15.57	15.41	15.40
RMSE		0.61	0.58	0.78	0.88
R^2^		0.95	0.95	0.91	0.89

RMSE, root mean square error (°C); R^2^, determination coefficient. The best results are underlined.

**Table 4:** Daily minimum temperature (°C) in Tordesillas (Valladolid) measured for 15 days (i.e., 1–15 June 2020), estimated with the neural architectures varying the number of neurons from four to one in the hidden layer (i.e., ANN (2-4-1), ANN (2-3-1), ANN (2-2-1) and ANN (2-1-1)), and fitting of the statistics.

Tordesillas	Data	ANN (2-4-1)	ANN (2-3-1)	ANN (2-2-1)	ANN (2-1-1)
1 June 2020	11.93	10.80	10.57	10.93	10.67
2 June 2020	13.67	13.38	13.54	13.27	12.85
3 June 2020	13.86	13.21	13.88	13.00	13.00
4 June 2020	11.33	9.32	9.37	9.20	9.16
5 June 2020	6.19	6.58	6.10	5.77	5.86
6 June 2020	9.59	9.84	9.75	9.70	9.00
7 June 2020	10.66	9.07	9.60	8.88	9.21
8 June 2020	7.8	7.31	7.41	6.38	6.54
9 June 2020	5.99	5.80	5.76	5.24	5.16
10 June 2020	7.67	6.72	5.84	5.66	5.98
11 June 2020	9.26	9.16	8.84	8.51	8.32
12 June 2020	8.66	8.13	8.21	8.45	7.72
13 June 2020	5.99	6.49	6.74	7.18	7.29
14 June 2020	7.19	7.19	6.94	7.07	7.07
15 June 2020	8.06	7.87	8.21	8.41	7.84
RMSE		0.83	0.88	1.11	1.12
R^2^		0.89	0.88	0.81	0.80

RMSE, root mean square error (°C); R^2^, determination coefficient. The best results are underlined.

## Nomenclature

ANN	Artificial Neural Network
ARRMSE	Average Relative Root Mean Square
BP-LM	Back-Propagation Levenberg–Marquardt algorithm
CGAN	Conditional Generative Adversarial Networks
DIDW	Dual Inverse Distance Weighting
ETo	Evapotranspiration
GAM	Generalized Additive Regression
GCM	General Circulation Models
GIS	Geographic Information System
IDW	Inverse Distance Weighting
IoT	Internet of Things
ITACyL	Agricultural Technological Institute in Castilla and León (Instituto Tecnológico Agrario de Castilla y León, in Spanish)
LR	Linear Regression
LSDV	Least Squares Dummy Variable
mamsl	meters above mean sea level
MARS	Monitoring Agriculture with Remote Sensing
MCYFS	MARS Crop Yield Forecasting System
MLP	Multilayer Feed-forward Perceptron
MLR	Multivariate Linear Regression
OCK	Ordinary Co-Kriging
OK	Ordinary Kriging
OLI	Operational land imager
RF	Random Forest
RMSE	Root Mean Square Error
R^2^	Coefficient of determination
SIAR	Agro-climatic Information System for Irrigation (Sistema de Información para el Asesoramiento al Riego, in Spanish)
SIDW	Semantics Inverse Distance Weighting
TIRS	Thermal infrared sensor
SIM	Spatial Interpolation Methods
VWS	Virtual Weather Station.

## Appendix A

Appendix A shows the information (altitude, latitude and longitude) of the 54 agrometeorological stations belonging to the Agro-climatic Information System for Irrigation (SIAR) InfoRiego [32], located in the nine provinces of Castilla and León Region, Spain, in Table A1.

**Table A1:** Location of the 54 agrometeorological stations belonging to the Agro-climatic Information System for Irrigation (SIAR) located in Castilla and León Region, Spain.

Province	Location	Altitude (mamsl)	Latitude (°)	Longitude (°)
Ávila	Nava de Arévalo	921	40.997	−4.765
Ávila	Muñogalindo	1128	40.597	−4.905
Ávila	Losar del Barco	1027	40.397	−5.535
Burgos	Valle de Losa	635	42.988	−3.220
Burgos	Condado de Treviño	551	42.719	−2.690
Burgos	Valle de Valdelucio	975	42.724	−4.081
Burgos	Lerma	840	41.987	−3.763
Burgos	Tardajos	770	42.353	−3.814
Burgos	Vadocondes	870	41.628	−3.573
Burgos	Santa Gadea del Cid	520	42.684	−3.108
León	Carracedelo	467	42.550	−6.733
León	Mansilla Mayor	791	42.512	−5.446
León	Cubillas de los Oteros	777	42.378	−5.511
León	Zotes del Páramo	779	42.265	−5.731
León	Quintana del Marco	750	42.201	−5.862
León	Hospital de Órbigo	835	42.463	−5.883
León	Bustillo del Páramo	874	42.439	−5.800
León	Sahagún	856	42.369	−5.006
León	Santas Martas	885	42.453	−5.362
Palencia	Torquemada	868	42.039	−4.300
Palencia	Villaeles de Valdavia	881	42.576	−4.558
Palencia	Villamuriel del Cerrato	750	41.952	−4.508
Palencia	Fuentes de Nava	744	42.090	−4.767
Palencia	Villoldo	817	42.256	−4.598
Palencia	Herrera de Pisuerga	821	42.549	−4.311
Palencia	Villaluenga de la Vega	927	42.525	−4.776
Palencia	Lantadilla	798	42.336	−4.300
Salamanca	Ciudad Rodrigo	635	40.618	−6.492
Salamanca	Arabayona	850	41.047	−5.393
Salamanca	Ejeme	812	40.769	−5.525
Salamanca	Aldearrubia	815	41.004	−5.493
Segovia	Gomezserracín	870	41.287	−4.299
Segovia	Navas de la Asunción	822	41.141	−4.486
Soria	Almazán	943	41.483	−2.556
Soria	Hinojosa del Campo	1043	41.743	−2.081
Soria	San Esteban de Gormaz	855	41.535	−3.220
Soria	Fuentecantos	1063	41.843	−2.434
Valladolid	Mayorga	748	42.172	−5.300
Valladolid	Finca Zamadueñas	714	41.626	−4.740
Valladolid	Medina del Campo	724	41.320	−4.904
Valladolid	Rueda	700	41.385	−4.968
Valladolid	Villalón de Campos	788	42.100	−5.034
Valladolid	Torrecilla de la Orden	793	41.219	−5.267
Valladolid	Olmedo	750	41.292	−4.717
Valladolid	Encinas de Esguevas	816	41.754	−4.091
Valladolid	Tordesillas	658	41.509	−4.989
Valladolid	Valbuena de Duero	756	41.662	−4.284
Valladolid	Medina de Rioseco	739	41.889	−5.030
Zamora	Colinas de Trasmonte	709	42.014	−5.821
Zamora	Villaralbo	659	41.497	−5.670
Zamora	Villalpando	701	41.825	−5.406
Zamora	Pozuelo de Tábara	714	41.780	−5.907
Zamora	Barcial del Barco	738	41.935	−5.644
Zamora	Toro	623	41.489	−5.470
